# Y chromosome AZFc microdeletion may have negative effect on embryo euploidy: a retrospective cohort study

**DOI:** 10.1186/s12920-023-01760-z

**Published:** 2023-12-11

**Authors:** Wei Jiang, Qijun Xie, Xin Li, Ye Yang, Ting Luan, Danyu Ni, Yuting Chen, Xinyu Wang, Chun Zhao, Xiufeng Ling

**Affiliations:** 1 Department of Reproductive Medicine, Nanjing Women and Children’s Healthcare Hospital, Women’s Hospital of Nanjing Medical University, 123 Tianfei Lane, Mochou Road, Nanjing, Jiangsu 210004 China; 2 Department of Obstetrics and Gynecology, Nanjing Women and Children’s Healthcare Hospital, Women’s Hospital of Nanjing Medical University, 123 Tianfei Lane, Mochou Road, Nanjing, Jiangsu 210004 China

**Keywords:** PGT-A, Y chromosome AZFc microdeletion, Embryo aneuploidy, Pregnancy outcome

## Abstract

**Background:**

Embryo aneuploidy is a main of principal reason of pregnancy loss, in vitro fertilization (IVF) failure and birth defects in offspring. Previous researchs have demonstrated that Y chromosome AZFc microdeletion was associated with reproduction outcomes, however, the relationship between Y chromosome AZFc microdeletion and embryo aneuploidy remains unexplored.

**Methods:**

This retrospective cohort study enrolled 513 patients with 603 cycles in the reproductive center of Nanjing Maternity and Child Health Care Hospital from January 1, 2016 to June 30, 2022. The study cohort was divided into two groups: the AZFc microdeletion group, comprising 53 patients and 58 cycles, and the control group, comprising 460 patients and 545 cycles. Statistical methods including restricted cubic spline and generalized estimating equation (GEE) were employed to evaluate the relationship between Y chromosome AZFc microdeletion and embryo euploidy.

**Results:**

294 and 2833 blastocysts were selected as AZFc microdeletion group and control group, respectively. Patients with Y chromosome AZFc microdeletion had significantly higher embryo aneuploid rate (33.0% vs. 27.3%, P < 0.05), lower rate of normal fertilization rate (81.5% vs. 90.3%, P < 0.05) and lower blastocysts formation rate (47.0% vs. 57.8%, P < 0.05) compared with the control group. However, no significant differences in pregnancy outcomes after euploid embryos transfer were observed between these two groups.

**Conclusions:**

Our study underscored the association between Y chromosome AZFc microdeletion and an elevated risk of embryo aneuploidy. Before the conventional intracytoplasmic sperm injection (ICSI) treatment, couples with Y chromosome AZFc microdeletion should be apprised of the heightened susceptibility to embryo aneuploidy. Preimplantation genetic testing for aneuploidy (PGT-A) should be introduced for selection.

**Supplementary Information:**

The online version contains supplementary material available at 10.1186/s12920-023-01760-z.

## Background

Embryo aneuploidy is the principal reason of pregnancy loss and in vitro fertilization (IVF) failure, while it is also responsible for birth defects in offspring [1, 2]. Preimplantation genetic testing for aneuploidy (PGT-A) is used to screen euploid embryos by trophectoderm (TE) biopsy, helping couples to avoid adverse pregnancy outcomes.

It is well known that the rate of aneuploid embryos increases dramatically with maternal age [3]. Several studies have assessed the relationship between embryo euploidy rates and ovarian stimulation process, culture conditions or the morphological features of blastocyst [4–6]. Although entire chromosomal aneuploidy in embryo originates mainly from meiotic errors during oocyte generation, approximately 8.1% of aneuploid embryos are still paternal in origin [7].

In terms of paternal factors, paternal chromosome abnormalities are the important causes of embryo aneuploidy, but it is unknown whether other male factors could lead to increased risk of embryo aneuploidy. Parameters such as paternal age, body mass index (BMI) and semen characteristics have been investigated in predictive models for aneuploid embryos [8–10].

While, limited attention has been devoted to exploring whether patients with specific chromosomal abnormalities, such as Y chromosome azoospermia factor (AZF) microdeletions, might had adverse effects on embryo euploidy. AZF is located on the long arm of the Y chromosome (Yq11), including three regions, AZFa, AZFb and AZFc, and AZFc microdeletion is the most frequent, which is among the best known genetic causes of male infertility. Several investigators have studied the prevalence of Y chromosome microdeletions in couples with recurrent pregnancy loss (RPL). Some studies have reported that the prevalence of Y chromosome microdeletions in AZF region was higher in men from RPL couples than control couples [11, 12]. However, other studies indicated no association between prevalence of Y chromosome microdeletion in AZF region and RPL [13–16]. Moreover, a recent study suggested that non-obstructive azoospermia (NOA) patients with AZFc microdeletions had lower rate of fertilization, clinical pregnancy rates (CPR), live birth rates (LBR) and cumulative LBR compared with NOA patients caused by other etiologies [17]. This leads us to question whether Y chromosome AZFc microdeletions could interfere with the normal physiology of the spermatocyte, increasing embryo aneuploidy rates.

The aim of this study was to investigate whether Y chromosome AZFc microdeletions correlate with embryonic euploidy of paternal origin. Furthermore, the pregnancy outcomes of Y chromosome AZFc microdeletions patients after euploid embryo transferred were also focused. This information would assist physicians to provide best medical care to patients with Y chromosome AZFc microdeletions.

## Methods

A retrospective cohort study was conducted in the reproductive center of Nanjing Women and Children’s Healthcare Hospital between January 1, 2016 and June 30, 2022, including 603 oocyte-retrieval cycles with PGT-A in 513 patients. Institutional Review Board approval was obtained for this study. All IVF cycles using PGT-A testing were included. We excluded patients with advanced age (≥ 38 years old) and those with single gene disorders or translocation.

All patients were assigned to one of two groups based on the indications for PGT: the AZFc microdeletion group or the control group. The AZFc microdeletion group included patients with Y chromosome AZFc microdeletion who wanted sex selection to avoid transmitting the deletion to their male descendants. The control group included patients with repeated implantation failure in IVF cycles and recurrent spontaneous abortion.

The primary outcomes were aneuploidy, euploidy and mosaicism. Secondary endpoints were blastocyst formation rate, clinical pregnancy rate and the rate of early pregnancy loss within each group.

### Ovarian stimulation

Controlled ovarian hyperstimulation for IVF included gonadotrophin releasing hormone (GnRH) agonist protocol, GnRH antagonist protocol, progestin primed ovarian stimulation (PPOS) protocol and mild stimulation protocol. We adjusted the doses of recombinant follicular stimulating hormone (rFSH, Gonal-F, Merck Serono, Italy) and urinary human menopausal gonadotropin (HMG Menopur, Ferring, Switzerland) according to the ovarian response, as monitored by ultrasound scan and sex hormone levels (FSH, luteinizing hormone, estradiol, and progesterone). Human chorionic gonadotrophin (hCG, Lizhu, China) at dose of 10,000 IU was used to trigger oocyte maturation when at least two follicles were measured 18 mm or more. Oocyte retrieval was scheduled 36 h after the trigger. All mature (metaphase II) oocytes were fertilized by intracytoplasmic sperm injection (ICSI). After ICSI, all fertilized oocytes were cultured in separate microdrops up to the blastocyst stage. Trophectoderm biopsy was performed on blastocysts with grades 4 or above and at least 1 score B for either ICM or TE, according to the Gardner criteria. All blastocysts were frozen by vitrification as per standard procedures.

### The detection of Y chromosome microdeletions

Y chromosome AZF microdeletion analysis was performed with a multiplex polymerase chain reaction (PCR) technique. Three different regions, AZFa, AZFb and AZFc, were analysed with six specific sequence-tagged site (STS) markers according to the recommendations of European Academy of Andrology (EAA) and European Molecular Genetics Quality Network (EMQN). The STS markers were as follows: sY84, sY86, sY127, sY134, sY254 and sY255. Sex-determining region of the Y chromosome (SRY) and zinc finger protein, X-linked (ZFX)/zinc finger protein, Y-linked (ZFY) were used as internal controls. Y chromosome microdeletions were detected in 53 patients, all of which were located in the AZFc region.

### PGT-A analysis

PGT-A was performed for all cycles using next genetic sequencing (NGS). DNA from all samples was amplified through SurePlex DNA Amplification System (Illumina, San Diego, CA, USA). Subsequently, amplified DNA was assessed for chromosome aneuploidy screening with a VeriSeq PGS Kit on a the MiSeq system (Illumina, San Diego, CA, USA) according to the manufacturer’s instructions. Only euploid embryos were allowed for transfer and all aneuploid or mosaic blastocysts were excluded from transfer.

### Frozen-thawed embryo transfer

In case of at least one euploid embryo was identified, single embryo transfer was performed. The first transplantation cycle for each patient was included. A total of 45 embryos were transferred in the Y chromosome AZFc microdeletions group and 351 embryos in the control group. Endometrial preparation and transfer procedures were chosen according to patients’ characteristics. A Serum HCG test was performed 14 days after the embryo transfer, and the vaginal ultrasound was done 28 days following the embryo transfer. HCG positivity referred to HCG levels of more than 5 IU/L. Clinical pregnancy was defined as the pregnancy diagnosed via ultrasonographic visualization of gestational sac in the uterus. Early pregnancy loss was defined as clinical pregnancy that were missed before the 12th week of pregnancy.

### Statistical analysis

Statistical analyses were performed using SPSS 27.0 software and R 4.2.1 statistical software. Continuous variables were expressed as mean with SD and compared between the two groups using Kruskal-Wallis test. Categorical variables were presented as n (%) and compared between the two groups using Pearson’s Chi-square tests or Fisher’s exact test. Restricted cubic spline was used to visualize the relation of maternal age with euploidy rate. To account for clustering among multiple embryos from the same couple, odds ratios (OR) and 95% confidence intervals (CIs) for the association between aneuploidy and Y chromosome AZFc microdeletions were estimated using logistic regression models with generalized estimating equations (GEE).The confounding factors included maternal age, paternal age, BMI, anti-müllerian hormone (AMH), methods of sperm retrieval, semen volume, sperm concentration, sperm motility, total gonadotropin dose and duration of ovarian stimulation. A P-value of < 0.05 was considered statistically significant.

## Results

A total of 603 IVF cycles involving 513 patients and 3,127 biopsied blastocysts were included for analysis. Among these, 294 blastocysts from 58 cycles in 53 patients were selected as AZFc microdeletion group, and 2833 blastocysts from 545 cycles in 460 patients were submitted as control group.

Table 1 showed the baseline characteristics for all cycles. The AZFc microdeletion group was significantly younger than the control group (*P* < 0.001). There were no differences in the maternal BMI, basal FSH level, basal estradiol (E_2_) level between the two groups. The level of AMH and duration of infertility were significantly higher in the AZFc microdeletion group, compared with the control group (*P* < 0.05). The number of previous pregnancies, previous live birth and previous pregnancy losses were significantly lower in the AZFc microdeletion group than the control group (*P* < 0.001). Besides, comparing with the control group, more patients obtained sperm from testicles epididymides and worse semen parameters including semen volume, sperm concentration, sperm motility, progressive motility and total sperm count in the AZFc microdeletion group (*P* < 0.001).

**Table 1:** Characteristics of the participants at baseline between the two groups

Variable	AZFc microdeletion	Control	*P* value
No. of cycles	58	545	
No. of patients	53	460	
Maternal age (years)	28.53 ± 2.82	32.00 ± 3.27	< 0.001
Paternal age (years)	30.14 ± 3.87	33.84 ± 4.91	< 0.001
Duration of infertility (years)	2.72 ± 2.09	2.24 ± 2.25	0.003
No. of prior pregnancies (%)			< 0.001
0	43 (74.1)	22 (4.0)	
≥1	15 (25.9)	523 (96.0)	
No. of prior live birth (%)			< 0.001
0	54 (93.1)	247 (45.3)	
≥1	4 (6.9)	298 (54.7)	
No. of prior clinical miscarriage (%)			< 0.001
0	45 (77.6)	32 (5.9)	
≥1	13 (22.4)	513 (94.1)	
Maternal BMI (kg/m^2^)	22.14 ± 3.08	22.08 ± 2.88	0.992
Basal FSH (mIU/mL)	7.39 ± 2.25	7.87 ± 2.44	0.113
Basal E_2_ (pg/mL)	44.31 ± 23.06	44.71 ± 24.24	0.908
AMH (ng/mL)	6.18 ± 4.27	4.51 ± 3.56	< 0.001
Methods of sperm retrieval (%)			< 0.001
Ejaculation	51 (87.9)	538 (98.7)	
Testicular/epididymal puncture	7 (12.1)	7 (1.3)	
Semen volume (mL)	1.89 ± 1.00	2.04 ± 1.72	< 0.001
Sperm concentration (*10^6^/mL)	10.36 ± 13.00	37.1 ± 15.56	< 0.001
Sperm motility (%)	16.1 ± 16.54	44.23 ± 13.98	< 0.001
Progressive motility (%)	10.5 ± 12.83	33.27 ± 12.39	< 0.001
Total sperm count (*10^6^)	22.86 ± 33.31	76.30 ± 71.80	< 0.001

Continuous variables presented as mean ± SD. Categorical variables presented as n (%)

Abbreviations: BMI, body mass index; E2, estradiol; FSH, follicle-stimulating hormone; AMH, anti-Müllerian hormone

A detailed comparison of cycle characteristics between the two groups were presented in Table 2. The total doses of gonadotropin, the duration of ovarian stimulation, the rate of metaphase II (MII) oocytes and the number of blastocysts were comparable between the two groups. The number of oocytes retrieved, MII oocytes and two distinct pronuclei (2PN) were higher in the AZFc microdeletion group than the control group, consistent with the baseline characteristics of cycles (*P* < 0.05). Whereas, normal fertilization rate and blastocysts formation rate were found to be lower in the AZFc microdeletion group compared with the control group (*P* < 0.001).

**Table 2:** Characteristics of controlled ovarian hyperstimulation cycles between the two groups

Variable	AZFc microdeletion	Control	*P* value
Total gonadotropin dose (IU)	2117.89 ± 489.59	2188.07 ± 554.03	0.280
Duration of ovarian stimulation (day)	9.21 ± 1.30	8.85 ± 1.54	0.070
No. of retrieved oocytes per cycle	14.33 ± 5.62	11.17 ± 5.61	< 0.001
No. of MII oocytes per cycle	13.33 ± 5.67	10.67 ± 5.53	< 0.001
The rate of MII oocytes (%)	773/821 (94.2)	5813/6089 (95.5)	0.095
No. of 2PNs per cycle	10.86 ± 4.83	9.63 ± 5.33	0.034
Normal fertilization rate (%)	630/773 (81.5)	5251/5813 (90.3)	< 0.001
No. of blastocysts per cycle	5.07 ± 3.42	5.48 ± 3.83	0.540
Blastocysts formation rate (%)	294/626 (47.0)	2989/5171 (57.8)	< 0.001

Continuous variables presented as mean ± SD. Categorical variables presented as n/N (%)

Abbreviations: PN, Primary nucleus

**Fig. 1:**
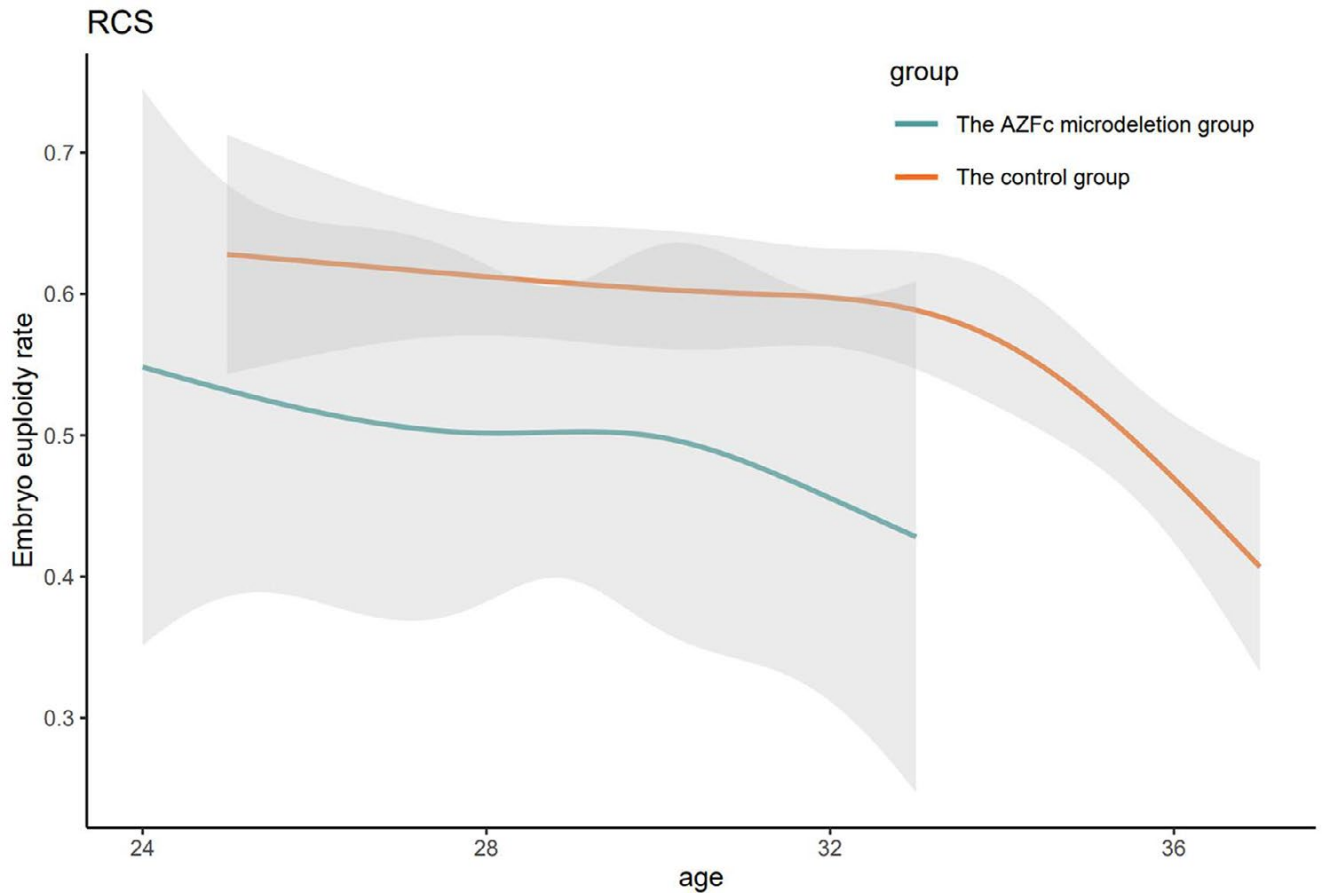
The restricted cubic splines for maternal age in association with the rate of embryo euploidy in the AZFc microdeletion group and the control group

The specific PGT-A outcomes and the outcomes of GEE analysis were described in Table 3. Of the 3,127 embryos, 3,009 embryos obtained successful PGT-A results (96.2%), including 95.9% of AZFc microdeletion group embryos and 96.3% of control group embryos. The AZFc microdeletion group demonstrated significantly higher rate of embryo aneuploidy compared with the control group (33.0% vs. 27.3%, *P* < 0.05). There was no significant difference in mosaic rate between the two groups (9.5% vs. 10.8%, *P* = 0.511). Logistic regression models using GEE was used to control potential confounders, and the results consistently showed a significant association between the AZFc microdeletion and embryo aneuploidy rate (OR: 1.654, 95% CI: 1.197–2.286, *P* < 0.01). Further analyzing the aneuploid embryos, difference of the rate of aneuploid embryos mainly reflected in duplication or deletion of entire chromosome (OR: 1.807, 95% CI: 1.227–2.660, *P* < 0.01). Moreover, the rate of embryos with monosomy X did not vary significantly by with and without the AZFc microdeletion (1.4% vs. 0.8%, OR: 1.635, 95% CI: 0.437–6.127, *P* = 0.465).The restricted cubic splines (RCS) incorporating linear regression models was built to evaluate the relationship between maternal age and the rate of embryo euploidy. The green fitted curve represents association of maternal age with the rate of embryo euploidy in the AZFc microdeletion group, and the orange fitted curve represents association in the control group. As shown in Fig. 1, the rate of embryo euploidy was significantly lower in the AZFc microdeletion group than the control group.

**Table 3:** Generalized estimating equation analysis for outcomes of PGT-A

Variable	AZFc microdeletion	Control	Adjusted OR(95% CI)	*P* value
No. of cycles	58	545	-	-
No. of patients	53	460	-	-
No. of blastocysts biopsied (%)	294	2833	-	-
No. of euploid embryos (%)	157 (53.4)	1650 (58.2)	0.676 (0.502–0.912)	0.010
No. of aneuploid embryos (%)	97 (33.0)	772 (27.3)	1.654 (1.197–2.286)	0.002
No. of entire chromosome aneuploid embryos (%)	61 (20.8)	481 (17.0)	1.807 (1.227–2.660)	0.003
No. of segmental aneuploid embryos (%)	32 (10.9)	225 (7.9)	1.235 (0.745–2.050)	0.413
No. of complex aneuploid embryos (%)	4 (1.4)	66 (2.3)	0.896 (0.309–2.598)	0.840
No. of mosaic embryos (%)	28 (9.5)	305 (10.8)	0.981 (0.579–1.661)	0.942
No. of uninformative embryos (%)	12 (4.1)	106 (3.7)	0.881 (0.421–1.841)	0.736
No. of embryos with monosomy X (%)	4 (1.4)	24 (0.8)	1.635 (0.437–6.127)	0.465

Categorical variables presented as n (%). CI = confidence interval

Adjusted: maternal age, paternal age, BMI, AMH, methods of sperm retrieval, semen volume, sperm concentration, sperm motility, total gonadotropin dose and duration of ovarian stimulation

The outcomes of the first euploid embryo transfer cycles were presented in Table 4. Totals of 45 and 351 patients transferred euploid blastocysts in the AZFc microdeletion group and control group, respectively. There was no significant difference in embryo developmental stage between the two group (*P* > 0.05). The maternal age was younger in the AZFc microdeletion group than the control group (*P* < 0.001). Furthermore, the AZFc microdeletion group showed lower proportion of good-quality embryo and thicker endometrial thickness on the days (*P* < 0.05). No difference was reported in the clinical pregnancy rate per patient between the AZFc microdeletion group and control group (*P* > 0.05). There was also no difference in the early pregnancy loss per clinical pregnancy (*P* > 0.05). Binary logistic regression analysis revealed no significant differences in pregnancy outcomes between the two groups (*P* > 0.05).

**Table 4:** Pregnancy outcomes after euploidy embryo transfer between the two groups

Variable	AZFc microdeletion	Control	*P* value
No. of frozen-thawed ET cycles	45	351	
Maternal age (years)	28.89 ± 2.78	31.92 ± 3.24	< 0.001
Embryo developmental stage (%)			0.743
D5	25 (55.6)	204 (58.1)	
D6	20 (44.4)	147 (41.9)	
Good-quality embryo transferring (%)	27 (60.0)	284 (80.9)	0.001
Endometrial thickness on ET day (mm)	9.14 ± 1.34	8.64 ± 1.65	0.012
Clinical pregnancy rate (%)	32/45 (71.1)	207/351 (59.0)	0.117
The rate of early pregnancy loss (%)	2/32 (6.3)	22/207 (10.6)	0.443

Continuous variables presented as mean ± SD. Categorical variables presented as n (%) or n/N (%). ET = embryo transfer

## Discussion

In this retrospective analysis, we observed a significant association between Y chromosome AZFc microdeletions and increased rates of embryonic aneuploidy, even after adjusting for potential confounding factors such as maternal age, paternal age, AMH, BMI, methods of sperm retrieval, semen volume, sperm concentration, sperm motility, total gonadotropin dose and duration of ovarian stimulation. Notably, this study did not find a heightened rate of embryos with monosomy X among men with AZF microdeletions. In addition, our results demonstrated lower normal fertilization rate and blastocysts formation rate in patients with Y chromosome AZFc microdeletions.

Until now, few researchers have evaluated the relationship between Y chromosome AZF microdeletions and embryonic aneuploidy of paternal origin. Some studies have reported significant increases in XY-disomic sperm in patients with Y chromosome microdeletions compared to oligozoospermia men without Y chromosome microdeletions [18–20]. For instance, Mateu assessed the incidence of numeric chromosomal abnormalities in spermatozoa and embryos from infertile patients with and without Y chromosome AZFc microdeletions, and found that compared with the patients without Y chromosome AZFc microdeletions, patients with Y chromosome AZFc microdeletions and high percentage of numeric chromosome abnormalities detected by fluorescence in situ hybridization (FISH) on sperm had significant increase of spermatozoa with diploidy for sex chromosome and higher incidence of chromosomally abnormal embryos, especially the sex chromosomally abnormal embryos [21]. Consistent with the finding reported by Mateu, in this study, a significant higher rate of aneuploid embryos was observed in patients with Y chromosome AZFc microdeletions. But our results can’t confirm Y chromosome microdeletions in the AZFc region is associated with embryos with monosomy X. This discrepancy may be due to that our data did not differentiate sperm FISH results. In addition, advances in IVF technology have led to a significantly lower rate of chromosomally abnormal embryos in our study compared to Mateu’s research, which includes embryos with monosomy X. This suggests that the differences in embryos with monosomy X between the two groups are narrowing, and more samples are needed to account for small differences.

The underlying mechanism for these findings remains unclear. Meiotic aneuploidies are mainly caused by abnormal segregation of homologous chromosomes in meiosis I or sister chromatids in meiosis II [22]. Segmental aneuploidies have complex origins, involving double-strand DNA breaks [23]. Our study has found the difference in embryo aneuploidy focused on entire chromosomal aneuploidy, which means meiotic progression of sperm in patients with Y chromosome AZFc microdeletions may be impaired. Previous studies have suggested that AZF region mutations is responsible for the meiotic abnormalities, with impairment of the synaptic process [24, 25]. Synapsis errors produce abnormal segregation of homologous chromosomes in meiosis I and generate spermatozoa with numerical chromosome abnormalities, such as aneuploidy or diploidy [26, 27]. Then the presence of aneuploidy sperm could lead to aneuploidy embryos ending in implantation failures or RPL.

Our results also suggested that Y chromosome AZFc microdeletions affect embryo quality, reducing normal fertilization rate, blastocyst formation rate and blastocyst score. Up to now, researches regarding effects of Y chromosomal microdeletions on pregnancy outcomes were still limited and had small samples size. Yu found that lower day 3 oocytes utilization rate, high-score embryo rate, lower cumulative CPR and cumulative LBR in patients with Y chromosome AZFc microdeletion, compared with non-obstructive azoospermia (NOA) patients with different etiologies [17]. The results were similar to Zhang’s report [28]. However, some studies insisted that AZF deletions had no adversely effects on embryo quality and clinical outcomes [29, 30]. In our study, the control group included the couple who suffered implantation failures or RPL, and thus had better spermic conditions than the control group in other studies, which just included men with azoospermia and severe oligozoospermia. This may explain the significant differences in embryo quality between the two groups in our study. As to pregnancy outcomes, our study all transferred euploid embryos, showing no differences between groups in clinical pregnancy rates and the rate of early pregnancy loss. In fact, embryonic chromosomal abnormalities are not the only cause of RIF and RPL. Though euploid embryo transfered, couples with RIF and RPL have worse prognosis compared with infertile couples in general. It may be more meaningful to study the outcomes of patients with Y chromosome microdeletions compared with the general population after euploid embryos transferred, especially on the basis of our finding that the embryos score was lower in patients with Y chromosome AZFc microdeletion.

The main limitation of the present study is that the genetic analysis technology for PGT-A does not allow us to distinguish the parental origin of embryonic aneuploidy. Meanwhile, this study is limited by retrospective design and small sample size. The incidence of Y chromosome AZF microdeletions in the population is very low, and only subset of patients who don’t hope to transmit the Y chromosome microdeletions to their male offspring use PGT-A for female sex selection [31]. Furthermore, it should be noted that this study is limited to the detection of microdeletions within the AZFc region. Additional large-scale randomized controlled trials are needed to confirm the conclusion of this study.

## Conclusions

In summary, this study provided evidence that patients with Y chromosome AZFc microdeletions exhibited reduced normal fertilization rate, reduced blastocyst formation rate and increased incidence of aneuploid embryos. These undesirable effects should be informed to patients with Y chromosome microdeletions, and PGT-A should be recommended for these patients to avoid risk of transferring aneuploid embryos.

### Electronic supplementary material

Below is the link to the electronic supplementary material.

Supplementary Material 1

## Data Availability

The data that support the findings of this study are available from the corresponding author upon reasonable request.
