# Supplementary material for: Y chromosome AZFc microdeletion may have negative effect on embryo euploidy: a retrospective cohort study
Source: BMC Med Genomics. 2023 Dec 11;16:324. doi: 10.1186/s12920-023-01760-z (PMC10712062; doi:10.1186/s12920-023-01760-z)
Supplement: Supplementary file 1 — Supplementary Material 1 [file 12920_2023_1760_MOESM1_ESM.docx]

Supplemental Table 1. Binary logistic regression analysis for pregnancy outcomes.

| **Variable** | **Adjusted OR(95% CI)** | ***P* value** |
| --- | --- | --- |
| Clinical pregnancy | 1.954 (0.935-4.086) | 0.075 |
| Early pregnancy loss | 0.415 (0.082-2.094) | 0.287 |

Adjusted: maternal age, BMI, embryo developmental stage, good-quality embryo transfer, endometrial thickness on embryo transfer day.
